# Increased propofol consumption with later anesthesia start times in sedated gastrointestinal endoscopy: insights from regression and machine learning models

**DOI:** 10.3389/fmed.2025.1670994

**Published:** 2025-10-21

**Authors:** Qiong Lan, Zhuonan Sun, Tian Wang, Zhuya Huang, Dengyang Han, Taotao Liu, Hua Zhang, Ye Wang, Rui Zhang, Binlong Li, Ning Yang, Yinyin Qu, Huili Liu, Mao Xu

**Affiliations:** ^1^Department of Anesthesiology, Peking University Third Hospital, Beijing, China; ^2^School of Electronic Engineering, Beijing University of Posts and Telecommunications, Beijing, China; ^3^Research Center of Clinical Epidemiology, Peking University Third Hospital, Beijing, China; ^4^Department of Gastroenterology, Peking University Third Hospital, Beijing, China

**Keywords:** propofol, temporal variations, sedated gastrointestinal endoscopy, manually-controlled administration, machine learning model

## Abstract

**Background:**

Chronopharmacology is an important but underexplored aspect of propofol administration. Despite the implementation of propofol administration models, none have yet incorporated temporal variables. This study aims to investigate the impact of temporal variations on propofol administration during sedated gastrointestinal (GI) endoscopy. Moreover, we aim to develop regression models to predict manually-controlled propofol administration that integrate temporal variables.

**Methods:**

This prospective single-center cohort study enrolled patients undergoing sedated GI endoscopy. For analysis, patients were categorized into 4 groups based on the anesthesia start time: Group 1 (8:00–10:00), Group 2 (10:00–12:00), Group 3 (13:00–15:00), and Group 4 (15:00–17:00). Perioperative characteristics and propofol doses were compared across groups. Correlation analysis was conducted to evaluate the relationship between propofol dose and the anesthesia start time. Subsequently, linear regression models were developed for manually-controlled propofol administration.

**Results:**

A total of 146 cases were included in the statistical analysis. Significant differences were found for all parameters related to propofol dose across the 4 different groups, including induction dose, maintenance dose, total dose and these doses per kilogram per hour. Furthermore, there were positive correlations between the anesthesia start time and all parameters. In the linear regression models, the induction dose equation incorporated the anesthesia start time, age and weight as variables. The model of the maintenance dose per kilogram per hour included the anesthesia start time, duration and weight as variables.

**Conclusion:**

The results suggest that propofol dose increases with later anesthesia start times. Therefore, further clinical administration of propofol should incorporate a heightened consideration of temporal factors.

**Trial registration:**

This prospective study has been registered in the Chinese Clinical Trial Registry (Registration date: December 3, registry number ChiCTR2400093328).

## Introduction

Over the past decade, there has been a substantial increase in the global population undergoing sedated examinations and procedures, including but not limited to sedated endoscopy, hysteroscopy, and oocyte retrieval. In China, about 7,000,000 patients undergo sedated gastrointestinal endoscopy GI annually (1). The advantages of sedated procedures, such as rapid recovery, enhanced comfort, and early discharge have significantly improved patient satisfaction and compliance (2, 3). Correspondingly, the precise administration of sedatives and anesthetics is crucial for optimizing procedural efficiency and patient turnover (4, 5).

Propofol is the most extensively applied anesthetic in sedated examinations and procedures (6). Various factors influence the dose of propofol, including age (7), gender (7), body weight (7, 8), circadian rhythm (9, 10), insomnia (11), anxiety (12), smoking (13), and others. Recently, the impact of circadian rhythm on propofol administration has attracted increasing attention (14).

Circadian rhythms are endogenous, entrainable oscillations of physiological structure and function that exhibit a periodicity of approximately 24 h. Studies have demonstrated that circadian rhythm influences propofol administration although these studies have limitations. For instance, in patients undergoing laparoscopic surgeries, the Narcotrend index, mean arterial pressure (MAP) and heart rate (HR) during procedures between 08:00 and 18:00 were observed to be higher than those during procedures between 22:00 and 5:00 (10). Another study demonstrated that patients undergoing elective laparoscopic abdominal surgeries between 8:00 and 12:00 required a higher dose of propofol compared to those undergoing surgeries between 18:00 and 22:00 (15). These studies compared surgeries conducted during daytime hours with those performed at night, thereby limiting their applicability. Another study illustrated that gynecological outpatient short-duration surgeries (abortion and curettage) performed between 8:30 and 11:30 required a lower dose of propofol compared to surgeries conducted between 14:00 and 17:00 (16). All these qualitative studies confirmed the temporal variation in propofol administration. However, because they lack quantitative computation, they were unable to offer practical guidance for clinical practice in propofol administration.

Therefore, this study aims to investigate the variations in propofol dose among patients undergoing sedated GI endoscopy during daytime hours and to construct a feasible model of propofol administration that includes circadian rhythm considerations. The anticipated outcomes are expected to facilitate faster patient turnover and recovery, as well as reduce adverse reactions among outpatients.

## Materials and methods

A prospective, single-center cohort study was conducted at the Endoscopic Ambulatory Surgery Center of Peking University Third Hospital from December 2024 to March 2025. The primary aim of this investigation was to determine whether temporal variations exist in the dose of propofol administered during sedated GI endoscopy. A secondary objective was to examine the variables influencing propofol dose in patients undergoing sedated GI endoscopy during daytime hours and to develop viable models for manually-controlled propofol administration that incorporate circadian rhythm considerations.

### Ethical statement

This study was conducted in compliance with the Helsinki Declaration and with the approval of the Ethics Committee at Peking University Third Hospital (Grant number M2024956). Furthermore, this prospective study has been registered in the Chinese Clinical Trial Registry (registry number ChiCTR2400093328).

### Study population

The study sought to recruit adult patients (18–65 years) with an American Society of Anesthesiologists (ASA) classification of I or II. ASA I patients were otherwise healthy except for the surgery, and ASA II patients had with mild systemic disease and good tolerance. Patients classified as ASA III and IV, who have severe systemic disease and substantive functional limitations or a constant threat to life, respectively, were not included. These patients underwent diagnostic esophagogastroduodenoscopy and colonoscopy under general anesthesia without intubation.

Informed consent was obtained from all patients prior to their inclusion in the study. Each participant was provided with a thorough explanation of the study’s objectives and nature and received a signed copy of the consent document.

The exclusion criteria were as follows: documented allergic reaction to propofol or opioids, history of opioids or propofol misuse, administration of general anesthesia within the preceding 30 days, the necessity for therapeutic procedures during GI endoscopy, anesthesia duration exceeding 60 min, incomplete colonoscopy due to inadequate bowel preparation, and unwillingness to participate in the research.

### Data collection

#### Pre-operative baseline collection

A total of 162 patients scheduled for GI endoscopy were recruited. Patients were required to undergo bowel preparation and were prescribed a laxative solution before GI endoscopy. The bowel preparation protocol applied in our hospital involved a divided administration method: half of the solution was taken the night before GI endoscopy, and the second half was taken early in the morning on the day of the procedure, between 2:00 to 7:00 a.m. The administration time of the second half of the solution depended on the specific situation of each patient.

Before GI endoscopy, patients received a preoperative interview to collect baseline data. This data included demographic information such as age, gender, height, and weight, as well as a medical history of chronic cardiovascular and cerebrovascular diseases, including hypertension, diabetes, cerebrovascular disease, hyperlipidemia, and coronary heart disease. Additionally, the interview gathered a history of other factors history that may affect propofol dosage, including depression, anxiety, insomnia, recent smoking, and alcoholism. GI endoscopy were conducted consecutively from 8:00 to 12:00 in the morning and 13:00 to 17:00 in the afternoon, except for a one-hour lunch break from 12:00 to 13:00.

#### Perioperative management

The administration of general anesthesia was conducted by two anesthesiologists (Q, L and Z, S), in accordance with institutional protocols and current guidelines (2, 3, 6). The intravenous catheterization was placed in the preparation room prior to GI endoscopy, followed by the infusion of Ringer’s lactate solution. Subsequently, prior to gastroscopy, dyclonine hydrochloride (10 mL: 0.1 g) was administered orally for mucosal anesthesia and lubrication.

Upon entry into the operating room and throughout the entire procedure, patients underwent continuous monitoring of HR, heart rhythm, oxygen saturation (SpO2), non-invasive blood pressure (NIBP), and the bispectral index (BIS). These parameters were recorded at 5 time points: before induction (T0), after the gastroscopy traversed the esophageal orifice (T1), after the gastroscopy passed through the cardia (T2), at the commencement of colonoscopy (T3) and before departure from the operating room (T4). Patients received oxygen via a nasal cannula at a flow rate of 5 L/min while positioned in the left lateral decubitus position.

The administration of propofol for GI endoscopy involved two phases: an initial bolus dose for induction, followed by a continuous infusion facilitated by a syringe pump. To determine the accurate dose, propofol administration adhered to a strategy of starting with a relatively low dose within the recommended range and gradually increasing it to meet clinical requirements (7, 17). Specifically, for induction, propofol was titrated to the onset of hypnotic effect with an infusion rate of 800 mL/h, alongside fentanyl at a dosage of 0.001 mg/kg (17). Following this, for maintenance, propofol was continuously infused at a rate of 4–5 mg/kg/h to maintain a BIS between 40 and 65 (18, 19). Additional doses of propofol (0.5–1 mg/kg) based on the extent was administered each time the patient exhibited a BIS ≥65 and/or physical reflexes, such as a gag reflex, coughing or body movement. If two consecutive supplementary doses of propofol were required, the maintenance rate of propofol was increased by 0.5 mg/kg/h (20). In cases when three administrations of propofol were insufficient to suppress the aforementioned reflexes, an additional dose of fentanyl was administered, and the patient was excluded from the data analysis to eliminate the confounding effect of varying fentanyl doses on propofol administration. The infusion rate of propofol for maintenance would be reduced by 0.5–1 mg/kg/h if BIS < 40.

All endoscopic procedures were carried out by experienced endoscopists who possess a minimum of 5 years of expertise in performing colonoscopies (21). Propofol infusion was discontinued immediately after the colonoscope reached the ileum and preparations for withdrawal began. Following removal of the endoscope from the anus, the attending clinician gently tapped patients on the shoulder and verbally prompted them to awaken. The awakening time was defined as the interval between the cessation of propofol infusion and the attainment of a modified Aldrete score >8. Once a modified Aldrete score of >8 was achieved, patients were transferred to the post-anesthesia care unit for further recovery.

The adverse reactions were defined as SpO2 below 90%, HR below 50 beats per minute (bpm), systolic blood pressure (SBP) below 90 mmHg or BIS below 40. When SpO2 dropped below 90%, we performed interventions such as the jaw-thrust maneuver, inserted an oropharyngeal or nasopharyngeal airway, or assisted ventilation via a facial mask according to the procedural requirements and the patient’s condition. In cases where the HR dropped below 50 bpm, atropine (0.5 mg) was administered (20) when SBP fell below 90 mmHg, ephedrine 5–10 mg was given and 200 mL Ringer’s lactate was also administered (20). The maintenance infusion rate of propofol would be reduced by 0.5–1 mg/kg/h if adverse reactions recurred after the initial event and intervention.

#### Sample-size estimation

The study was designed to compare induction and maintenance doses across 4 distinct periods: Group 1 (8:00–10:00), Group 2 (10:00–12:00), Group 3 (13:00–15:00), and Group 4 (15:00–17:00), categorized by anesthesia start time. Due to clinical constraints, however, the distribution of participants among different groups was uneven, with a higher number of patients allocated to Group 1 and Group 3. In our pilot study, the maintenance doses per kilogram per hour were 6.35 ± 2.09 mg/kg/h and 7.51 ± 2.00 mg/kg/h (*n* = 10) for Groups 1 and 3, respectively. To achieve statistical significance with a two-sided *α* value of 0.05 and 85% power, while accounting for a 10% drop-out rate, we determined that a sample size of 55 participants was necessary in each of Groups 1 and 3.

### Statistical analysis

The statistical analysis was performed using SPSS 25.0 software. The Kolmogorov–Smirnov test was used to assess the normality of the data distribution. Categorical variables were presented as numbers and percentages, while continuous variables were reported as mean ± standard deviation (SD) or median ± interquartile range (IQR). A one-way analysis of variance (ANOVA) was used to compare the 4 groups differentiated by varying initial times of anesthesia induction, followed by the Least Significant Difference (LSD) test for post-hoc pairwise comparisons. For categorical variables, the *χ*^2^ test or the Fisher’s exact test was applied. Statistical significance was set at a two-sided *p*-value < 0.05.

The correlations between the anesthesia start time and all 6 parameters of propofol dosage were analyzed using linear regression. The association between the number of patients requiring supplemental administration of propofol and the anesthesia start time was examined using a two-way ordered chi-square test with Kendell’s tau-b.

To develop linear regression models for manually-controlled propofol administration, the dataset was divided into constructing and testing subsets using a random 80%:20% split. The constructing subset was used to develop the predictive model, while the testing subset was used to evaluate the model’s performance in predicting propofol administration. For the regression analysis, univariate linear regression was initially employed to identify potential risk factors for the induction dose and maintenance doses per kilogram per hour. All candidate variables with a *p*-value <0.2 were subsequently included in multiple linear regression analysis. These candidate variables were evaluated for collinearity and, if collinearity was present, the variables were selected according to clinical significance. Following this, multivariable logistic regressions were performed to further analyze the data.

### Machine learning regression

The dataset was also divided into training and testing subsets using a random 80%:20% split. Two separate neural networks were constructed for predicting the induction dose and the maintenance dose per kilogram per hour, respectively.

For the output induction dose, a neural network comprised of 3 layers was constructed: the first layer contained 64 neurons, the second layer 32 neurons, and the last layer produced a single output value. The Rectified Linear Unit (ReLU) was used as the activation function, and a 20% dropout layer was incorporated after the first two layers to mitigate overfitting. The Adam optimizer was used for model training, with a learning rate of 0.001 and L2 regularization (weight decay = 1e-5).

To predict the maintenance dose per kilogram per hour, a neural network comprised of 4 layers was constructed. The first layer consisted of 128 neurons; the second layer, 64 neurons; the third layer, 32 neurons; and the final layer produced a single output value. The ReLU activation function was applied after each fully connected layer to introduce nonlinearity. Additionally, a dropout rate of 0.01 was implemented after the first and second layers to reduce overfitting. The RMSprop optimizer was used for parameter optimization, with a learning rate of 0.005, and L2 regularization (weight decay = 1e-4).

Both models were trained over a total of 300 epochs, using the mean squared error (MSE) as the loss function. The training loss was recorded at each epoch. An early stopping strategy was applied based on the validation set MSE. If the validation loss did not decrease for several consecutive epochs, training was terminated early, and the model parameters with the best validation performance were saved.

### Comparison of the predictive performance metrics among regression models

The predictive performances of the multiple linear and machine-learning models were compared using mean absolute error (MAE), root mean square error (RMSE), and the coefficient of determination (*R*^2^). The optimal model is characterized by the highest *R*^2^ and lowest errors (22).

## Results

### Demographic characteristics and perioperative conditions

**Figure 1:**
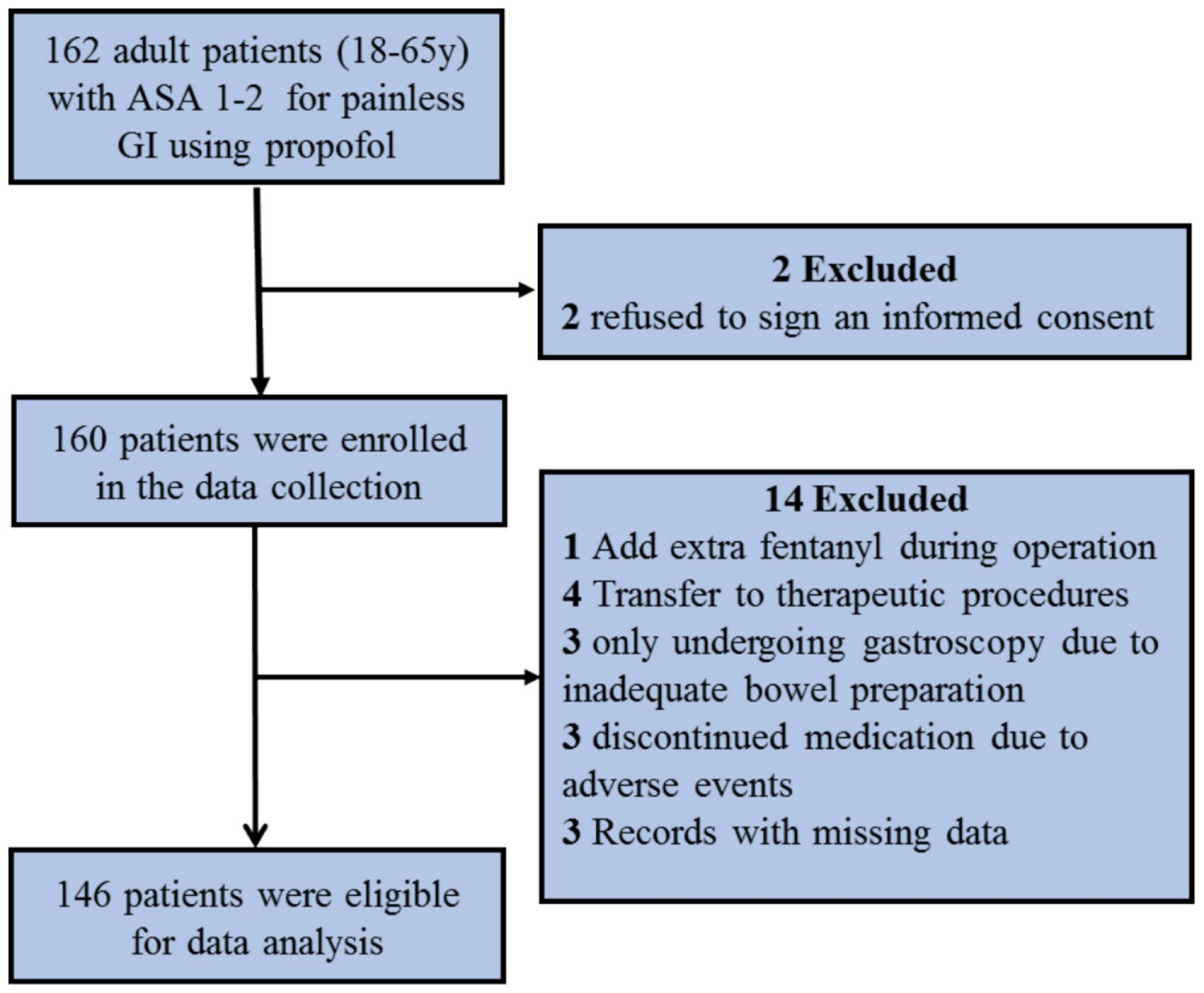
Study flow chart.

A total of 162 patients were initially screened for the study. Ultimately, 146 patients were eligible for data analysis (Figure 1). To determine whether variations exist in the dosage of propofol during sedated GI endoscopy at different time intervals, the data were categorized into four groups for analysis, based on the anesthesia start time: Group 1 (8:00–10:00), Group 2 (10:00–12:00), Group 3 (13:00–15:00), and Group 4 (15:00–17:00). The demographic and baseline characteristics of the patients are shown in Table 1. No significant differences in baseline characteristics were observed across the different groups.

**Table 1:** Demographic and baseline characteristics.

Variables	Group 1 (*n* = 53)	Group 2 (*n* = 25)	Group 3 (*n* = 57)	Group 4 (*n* = 11)	*F/T*	*P*
Age, yrs	44.55 ± 10.70	47.12 ± 12.33	44.95 ± 12.30	39.64 ± 9.62	1.08	0.36
Gender, Female	33 (62.26%)	12 (48%)	31 (54.36%)	5 (45.45%)	2.09	0.56
Height, cm	166.8 ± 7.56	167.1 ± 8.64	166.7 ± 7.63	168.9 ± 8.58	0.26	0.85
Weight, kg	65.15 ± 12.91	63.52 ± 9.97	64.29 ± 13.07	68.91 ± 14.78	0.52	0.67
Body Mass Index (BMI)	23.3 ± 3.65	22.72 ± 2.79	22.97 ± 3.42	24.03 ± 3.94	0.45	0.72
ASA classification, II	8(15.1%)	2(8%)	10(17.9%)	1(9.1%)	0.71	1.00
Chronic diseases*	6 (11.32%)	1 (4.0%)	7 (12.28%)	1 (9.1%)	1.30	0.77
Other factors^†^	8 (15.09%)	2 (8.0%)	3 (5.26%)	2 (18.2%)	4.02	0.22

*Chronic diseases include cardiovascular and cerebrovascular diseases, such as hypertension, diabetes, cerebrovascular disease, hyperlipidemia, and coronary heart disease.

^†^Other factors refer to additional factors that may impact propofol dosage, as indicated by previous studies, including insomnia, depression, anxiety, current smoking and alcoholism consumption.

Hemodynamic parameters (HR, SpO2, BIS, SBP, and MAP) and adverse reactions are detailed in Supplementary Material 1 and Table 2, respectively. No significant differences in hemodynamic parameters (Supplementary Material 1) or adverse reactions (Table 2) were observed across groups except for the baseline SBP measured prior to the anesthesia. These results indicate a comparable depth and efficacy of anesthesia among the groups.

**Table 2:** Hemodynamic adverse reactions during anesthesia.

Variables	Group 1 (*n* = 53)	Group 2 (*n* = 25)	Group 3 (*n* = 57)	Group 4 (*n* = 11)	*F/T*	*P*
SBP < 90	5(9.4%)	3(12%)	11(19.3%)	0	3.52	0.29
SpO2 < 90	1 (1.89%)	1 (4%)	1 (1.75%)	0	1.37	0.68
HR < 50	1(1.9%)	2 (8%)	2(3.5%)	0	2.09	0.60
BIS<40	0	0	5 (8.77%)	0	5.70	0.07

### Propofol dosage increased with a later initial time of anesthesia induction

To determine the temporal variation in propofol dosage, we analyzed the duration and dosage of propofol administration, as well as the awakening time after the cessation of propofol infusion (Table 3). The results indicated no significant differences in the duration of propofol infusion or the awakening time after cessation of propofol infusion (Table 3). Together with the absence of significant differences in hemodynamic parameters and adverse reactions, as previously noted, these results suggest comparable GI endoscopy and anesthesia efficacy across different time groups.

**Table 3:** Intraoperative condition of propofol administration.

Variables	Group 1 (*n* = 53)	Group 2 (*n* = 25)	Group 3 (*n* = 57)	Group 4 (*n* = 11)	*F*	*P*
Duration of propofol infusion, min	17.95 ± 4.80	16.36 ± 4.84	17.71 ± 4.42	17.63 ± 6.90	0.65	0.59
Awakening time after cessation of propofol infusing, min	5.19 ± 2.41	4.64 ± 2.11	5.18 ± 2.92	6.00 ± 2.83	0.22	0.88
Total dose, mg	202.1 ± 45.29	209.7 ± 49.59	231.7 ± 55.69	232.9 ± 62.08	3.53	0.001
Total dose per kilogram per hour, mg/kg/h	10.99 ± 2.48	13.34 ± 5.86	12.87 ± 3.47	12.65 ± 3.81	3.32	0.006
Induction dose, mg	83.30 ± 18.24	85.04 ± 23.64	93.84 ± 25.33	102.3 ± 16.87	3.444	**<0.001**
Induction dose per kilogram, mg/kg	1.29 ± 0.31	1.35 ± 0.35	1.48 ± 0.37	1.51 ± 0.31	5.371	**0.001**
Maintenance dose, mg	119.4 ± 40.24	124.7 ± 45.80	138.6 ± 46.48	129.3 ± 52.16	2.92	**0.035**
Maintenance dose per kilogram per hour, mg/kg/h	6.30 ± 1.81	7.22 ± 2.91	7.61 ± 2.51	6.54 ± 1.61	4.97	0.002

Bold values indicate predictors with statistical significance *p* < 0.05).

**Figure 2:**
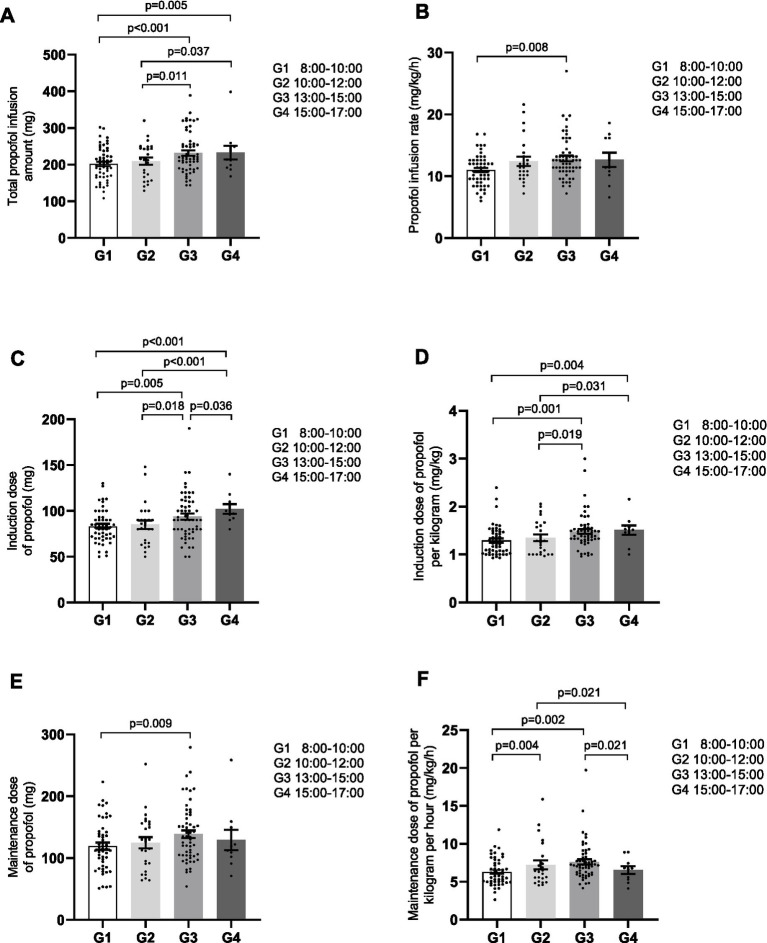
Parameters of propofol dose trends to increase with later anesthesia start times. The parameters include: **(A)** total propofol infusion dose; **(B)** propofol infusion rate; **(C)** induction dose of propofol; **(D)** induction dose of propofol per kilogram; **(E)** maintenance dose; **(F)** maintenance dose per kilogram per hour.

**Figure 3:**
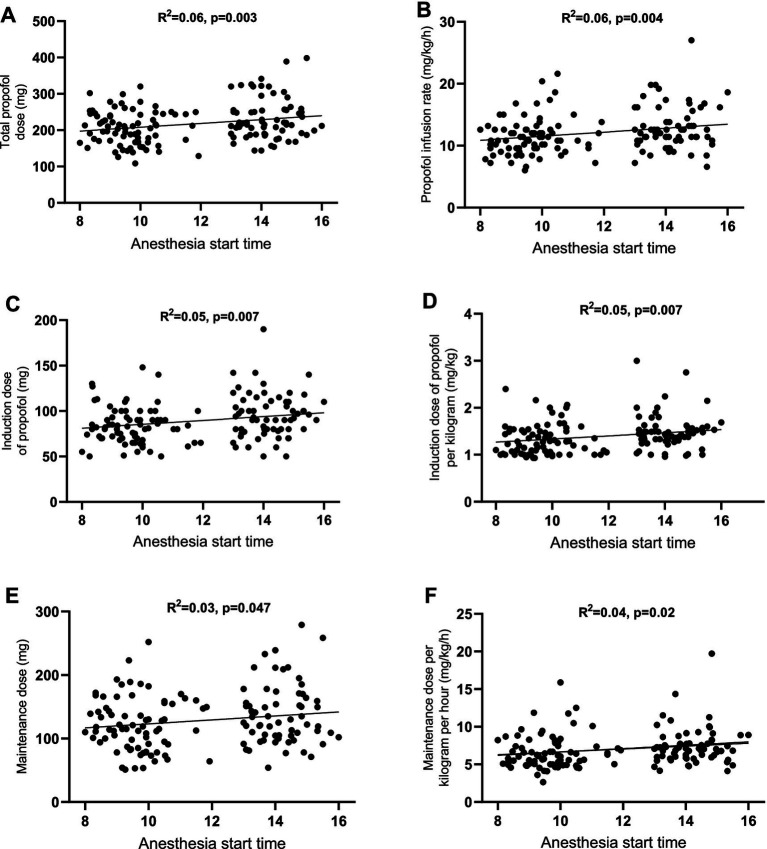
A positive correlation between propofol dosage and the anesthesia start time. The data discontinuity observed between 12:00–13:00 corresponded to the scheduled lunch break. **(A)** Total propofol dose; **(B)** propofol infusion rate; **(C)** induction dose of propofol; **(D)** induction dose of propofol per kilogram; **(E)** maintenance dose; **(F)** maintenance dose per kilogram per hour.

Next, we compared the dose of propofol across different time groups. Significant differences were observed among the groups for all 6 parameters of propofol dosage: total dose, propofol dose per kilogram per minute, induction dose, induction dose per kilogram, maintenance dose, and maintenance dose per kilogram per hour (Table 3). Pairwise comparisons revealed that more pronounced differences were found between groups with larger intervals in anesthesia start time while smaller differences were observed between groups with adjacent anesthesia start time. For example, all 6 parameters were significantly higher in Group 3 compared to Group 1 (Figures 2A–F), and 3 parameters were significantly higher in Group 4 compared to Group 1 (Figures 2A,C,D). When comparing Group 2 with Group 3 (Figures 2A,C,D) or Group 4 (Figures 2A,C,D), significant differences were observed in 3 parameters. One parameter demonstrated significant differences when comparing Group 1 with Group 2 (Figure 2F) or Group 3 with Group 4 (Figure 2C). These results indicate an increasing trend in propofol dosage with later anesthesia start times.

To confirm the correlation between the anesthesia start time and propofol dose, correlation analysis was conducted. The results demonstrated a positive correlation between the anesthesia start time and all six parameters of propofol dose: total propofol dose (*r*^2^ = 0.06, *p* = 0.003) (Figure 3A), propofol dose per kilogram per hour (*r*^2^ = 0.06, *p* = 0.004) (Figure 3B), induction dose (*r*^2^ = 0.05, *p* = 0.007) (Figure 3C), induction dose per kilogram (*r*^2^ = 0.05, *p* = 0.007) (Figure 3D), maintenance dose (*r*^2^ = 0.03, *p* = 0.047) (Figure 3E), and the maintenance dose of propofol per kilogram per hour (*r*^2^ = 0.04, *p* = 0.02) (Figure 3F).

### The percentage and frequency of patients requiring supplementary dosing increased with later anesthesia start time

**Figure 4:**
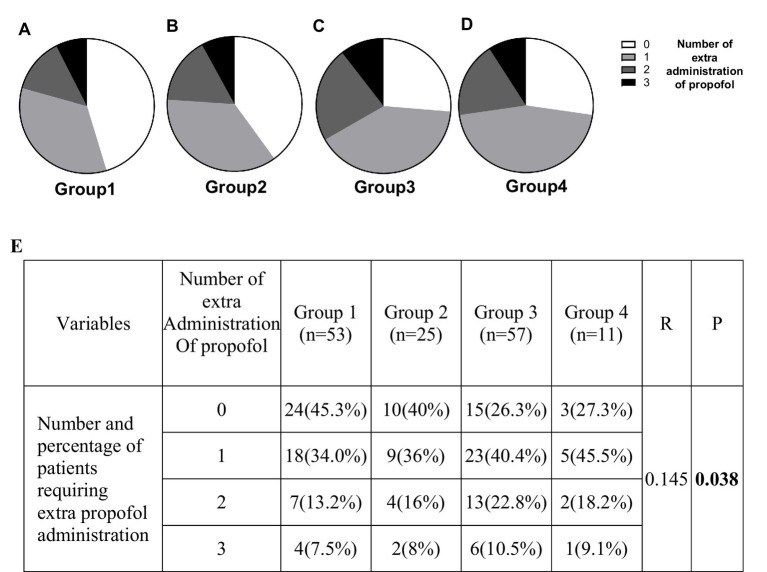
The percentage and frequency of patients requiring extra propofol doses increased with a later anesthesia start time. Values of 0, 1, 2, and 3 represent the number of additional propofol doses required. Panels **(A–D)** represent Groups 1, 2, 3, and 4, respectively. Panel **(E)** presents the number and percentage of patients requiring additional doses among the four groups.

Our propofol administration strategy, initiating with a relatively low dose and gradually increasing it, facilitated accurate propofol delivery and enable the investigation of propofol dose, although it was accompanied by increased BIS and/or physical responses. Among 146 patients, 95 (65.1%) required additional propofol administration. Specifically, 55 patients (57.9%) required one additional administration of propofol, 27 patients (28.4%) required two, and 13 patients (13.7%) required three. Group 1 had the highest percentage of patients who did not require extra dosing and the lowest percentage of patients who required 3 times of extra dosing. Later anesthesia start times, the percentage and frequency of patients requiring extra dosing of propofol increased (*p* = 0.038) (Figure 4). The mean dose of supplementary administration did not demonstrate statistical significance among the 4 groups (Supplementary Material 2).

### The multiple linear regression models exhibited a positive correlation between the start time and propofol dose

Previous studies have developed models to predict manually-controlled propofol administration in GI endoscopy (23). However, to our knowledge, no prior study has examined the impact of circadian rhythm on these regression models (24). Our results confirmed the influence of the anesthesia start time on propofol administration. We subsequently developed 2 sets of regression models for manually-controlled propofol administration that incorporated this variable. Based on the practice of manually-controlled propofol administration, the models predicted two dependent variables: the induction dose and the maintenance dose per kilogram per hour.

**Figure 5:**
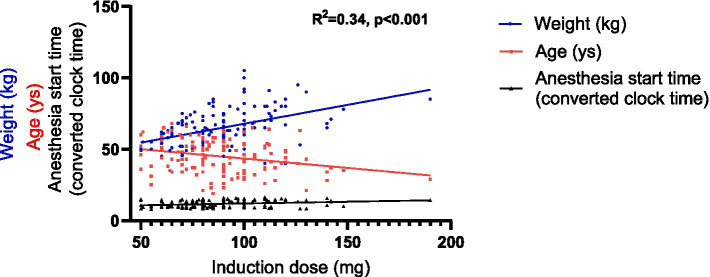
Graphical representation of the linear regression model of the induction dose. The anesthesia start time, originally recorded as hh:mm, was converted into decimal hours using the following formula: decimal time = hh + (mm/60).

First, linear regression models were developed using SPSS. For the induction dose, a univariable linear regression analysis was performed (Supplementary Material 3), followed by an assessment of collinearity (Supplementary Material 4). The analysis identified 5 impact factors for inclusion in the multiple linear regression model: age, gender, weight, the anesthesia start time and the presence of chronic diseases. Consequently, the multiple linear regression was represented by the following equation: Induction dose per kilogram = 53.113–0.447 × Age+ 0.656 × Weight + 2.003 × The anesthesia start time (*R*^2^ = 0.34, *p* < 0.001) (Table 4 and Figure 5).

**Table 4:** Multiple linear regression for the induction dose as the dependent variable.

Variables	Unstandardized coefficients		*t*	*p*
	*B*	Std. error		
**Age**	−0.447	0.138	−3.227	**0.002**
Gender	−6.575	4.369	−1.505	0.135
**Weight**	0.656	0.171	3.838	**<0.001**
**Anesthesia start time**	2.003	0.665	3.011	**0.003**

Bold values indicate predictors with statistical significance *p* < 0.05).

**Figure 6:**
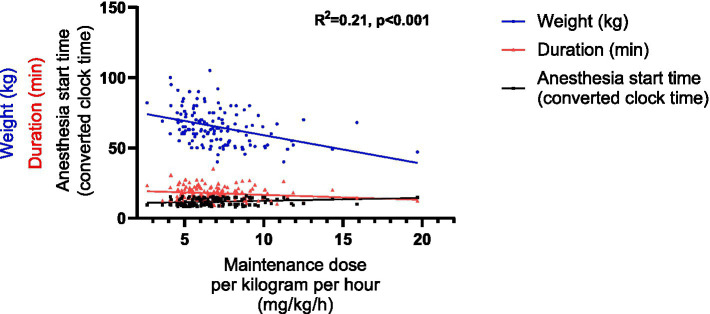
Graphical representation of the linear regression model of the maintenance dose per kilogram per hour. The anesthesia start time, originally recorded as hh:mm, was converted into decimal hours using the formula: decimal time = hh + (mm/60).

For the maintenance dose per kilogram per hour, 6 impact factors were incorporated into the multiple linear regression model based on the univariable linear regression (Supplementary Material 5) and the collinearity analysis (Supplementary Material 6). These factors included gender, weight, other relevant factors affecting propofol dose, the anesthesia start time, duration and the induction dose per kilogram. Consequently, the multiple linear regression model was represented by the following equation: Maintenance dose per kilogram per hour = 11.455–0.074 × Weight −0.076 × Duration + 0.172 × The anesthesia start time (*R*^2^ = 0.21, p < 0.001) (Table 5 and Figure 6).

**Table 5:** Multiple linear regression for the dependent variable of maintenance dose per kilogram per hour.

Variables	Unstandardized coefficients		*t*	*p*
	*B*	Std. error		
Gender	−0.279	0.505	−0.552	0.582
**Weight**	−0.074	0.021	−3.572	**<0.001**
Other factors	−0.519	0.613	−0.846	0.399
**Anesthesia start time**	0.172	0.078	2.209	**0.029**
**Duration**	−0.076	0.038	−1.996	**0.048**
Induction dose per kilogram	0.119	0.566	0.210	0.834

Bold values indicate predictors with statistical significance *p* < 0.05).

### Machine learning regression models

Machine learning regression models were also developed. Extensive model experiments were performed (Supplementary Material 7). Given the volume of data and analysis requirements, the selected models demonstrated optimal suitability for this study by exhibiting lower errors and higher *R*^2^ values. For predicting the induction dose, the model’s input features included: age, gender, height, weight, ASA classification, presence of any chronic diseases, other relevant factors and the time of anesthesia induction. For predicting the maintenance dose per kilogram per hour, the input features of the predictive model included: age, gender, BMI, ASA classification, presence of any chronic diseases, other relevant factors, duration and the time of anesthesia induction. The statistical parameters of the models (RMSE, MAE, *R*^2^) were presented in Table 5. The feature importance and the training loss curves were illustrated in Supplementary Material 8.

### Comparison of the linear regression models and machine learning regression models

The linear regression models were compared with the machine learning regression models. For predicting induction dose, the linear regression model demonstrated enhanced predictive performance compared to the machine learning model, characterized by lower RMSE, MAE and higher *R*^2^. Conversely, for predicting the maintenance dose per kilogram per hour, the machine learning model demonstrated superior performance compared to the linear regression model based on RMSE, MAE, and *R*^2^ parameters (Table 6).

**Table 6:** Comparison of the linear regression models and machine learning regression models.

Model	Outputs	RMSE	MAE	*R* ^2^
Linear regression model	Induction dose	21.60	17.90	0.34
	Maintenance per kilogram per hour	2.36	1.71	0.21
Machine learning algorithm	Induction dose	24.13	18.14	0.28
	Maintenance per kilogram per hour	1.61	1.15	0.37

RMSE, root mean square error; MAE, mean absolute error; *R*^2^, the coefficient of determination.

## Discussion

Chronopharmacology represents a crucial yet insufficiently explored dimension of propofol administration. Previous studies have investigated the influence of circadian rhythm on propofol administration; however, these studies had limitations, such as small sample sizes and lack of clear clinical practice (10, 15, 16). This cohort study prospectively collected data from 162 cases during sedated GI endoscopy using propofol, confirmed the temporal variation in propofol administration, and developed regression models for manually-controlled propofol administration in these contexts.

Previous researches have indicated that under target-controlled infusion (TCI) of propofol, BIS was significantly lower in the nocturnal group (22:00–2:00) compared to the diurnal group (8:00–12:00) (25). Furthermore, when BIS values were maintained at comparable levels, a greater dose of propofol was required during daytime administration than at nighttime 10. Nonetheless, these studies offer limited clinical guidance, as the majority of surgeries are conducted during daytime hours. There is a paucity of research examining temporal variations in propofol administration during the day. One study indicated that compared with short-duration gynecological procedures conducted during 8:30–11:30, propofol dose was higher in procedures undergoing during 14:00–17:00 (16). Our study demonstrates that, within a comparable population and consistent GI endoscopy stimulation across different anesthesia start time, the dose of propofol increased with later anesthesia start times, consistent with previous research.

Chronopharmacology can be divided into two primary areas: chronopharmacokinetics and chronopharmacodynamics. In the context of pharmacokinetics, propofol undergoes hepatic metabolism (17). The hepatic blood flow (26) and cytochrome P450 monooxygenase (5, 27), the enzyme responsible for the metabolism of numerous drugs including propofol, exhibit circadian fluctuations. Studies have shown that P450 monooxygenase activity increased at night and decreased during the day in a rat model (28). These results suggest that the pharmacokinetics of propofol exhibit a circadian rhythm. Within the realm of pharmacodynamics, the central nervous system depression induced by propofol is mediated through gamma--aminobutyric acid (GABA) (17, 29) on GABAergic neurons. Studies have shown that the morphology and function of neurons are subject to circadian rhythm. For instance, variations in the dendritic structure and spine density of neurons in the rat frontal cortex have been observed to follow circadian patterns (30, 31). Another study showed that GABAergic activity peaked at nocturnal hours and reached a trough at diurnal hours in the cerebral cortex of Syrian hamsters (32). These studies support the impact of circadian rhythm on neuronal activity targeted by propofol.

To our knowledge, no existing models of propofol administration, whether manually-controlled or TCI, have been developed that incorporate circadian rhythm factors. In this study, linear regression models and machine learning algorithms were developed to facilitate manually-controlled propofol administration. The linear regression model for induction dose included age, weight and the anesthesia start time as influencing factors, with age and weight exerting a more substantial impact on propofol dosage than the anesthesia start time. However, age was not included in the model of the maintenance dose per kilogram per hour. In that model, the duration exerted a relatively smaller influence compared to the anesthesia start time. These models align with the clinical practice and experience.

According to expert consensus (7, 33) and the authors’ experience, anesthesiologists consider age as a crucial factor influencing propofol induction. Consequently, they tend to select a lower induction dose per kilogram within the recommended range (1.5–2.5 mg/kg) for elderly patients. However, age appears to be a less critical consideration when determining the maintenance dose per kilogram per hour. Regarding the maintenance dose, it is widely accepted that, due to the redistribution of propofol, the maintenance dose decreases as the duration of administration extends (17). These findings were all consistent with our models. However, the influence of circadian rhythm on propofol dosing has not been thoroughly investigated before. Our models indicate that the timing of anesthesia induction exerts a more significant impact than the duration of administration. These results underscore the need to consider circadian rhythm in determining propofol dosing.

However, this study has certain limitations. First, due to clinical constraints, our data collection was restricted to specific time periods. Previous animal studies have indicated that anesthetic pharmacodynamics may adhere to a Cosine pattern rather than a linear model when data are collected over 24 h. For instance, one rat study demonstrated that the longest pharmacological effect (the loss of righting reflex) of propofol injection occurred shortly afternoon during the rest phase and the shortest effect shortly after midnight during the active phase (9). In our study, the maintenance dose of propofol per kilogram per hour during 15:00–17:00 was lower than that during 13:00–15:00, which seems to be consistent with the former study. To determine whether propofol administration follows a cosine pattern, further studies are required.

Additionally, the models were developed from data from patients undergoing sedated GI endoscopy. The applicability of these models to anesthetic management and surgeries exceeding 1 h in duration requires further investigation. Nonetheless, the present models provide a quantitative framework for propofol dosing in sedated GI endoscopy and highlight the potential influence of circadian rhythm on drug requirements.

Another limitation may involve sleep deprivation induced by the bowel preparation protocol, which required patients to ingest the second solution early in the morning, potentially disrupting sleep. Although animal studies suggest that sleep deprivation can alter propofol consumption (34, 35), this has not been confirmed in human clinical studies. Future controlled trials are needed to isolate this potential confounding factor.

Finally, due to the limited sample size in Groups 2 and 4, some parameter estimates may be underpowered. That said, the primary conclusions of this study are supported by other inter-group comparisons with sufficient statistical power (all >0.8), indicating that the overall findings are robust.

## Data Availability

The datasets presented in this study can be found in online repositories. The names of the repository/repositories and accession number(s) can be found in the article/Supplementary material.
