# Supplementary material for: Increased propofol consumption with later anesthesia start times in sedated gastrointestinal endoscopy: insights from regression and machine learning models
Source: Front Med (Lausanne). 2025 Oct 21;12:1670994. doi: 10.3389/fmed.2025.1670994 (PMC12583158; doi:10.3389/fmed.2025.1670994)
Supplement: Supplementary file 1 [file Table_1.docx]

**Supplementary 1. Hemodynamics parameters.**

| **Variables** | **Time point** | **Group 1(n=53)** | **Group 2**  **(n=25)** | **Group 3**  **(n=57)** | **Group 4**  **(n=11)** | **F/T** | **P** |
| --- | --- | --- | --- | --- | --- | --- | --- |
| **SBP** | T0 | 132.7±19.09 | 142.7±17.13 | 125.1±14.86 | 124.0±10.23 | 2.85 | 0.04^*^ |
|  | T1 | 121.7±19.01 | 112.1±17.40 | 119.9±21.02 | 116.5±8.36 | 1.11 | 0.35 |
|  | T2 | 112.7±13.81 | 108.8±18.49 | 109.9±16.51 | 103.8±14.18 | 0.72 | 0.54 |
|  | T3 | 104.9±10.02 | 100.9±12.01 | 103.2±14.41 | 107.3214.18 | 0.69 | 0.56 |
|  | T4 | 105.1±13.66 | 107.4±13.28 | 104.8±14.71 | 105.1±9.60 | 0.15 | 0.93 |
| **MBP** | T0 | 97.92±14.02 | 101.6±16.59 | 96.59±14.13 | 94.85±8.38 | 0.34 | 0.80 |
|  | T1 | 90.01±14.33 | 82.64±12.89 | 88.44±15.27 | 86.17±5.42 | 1.17 | 0.32 |
|  | T2 | 81.26±18.28 | 78.38±14.10 | 81.17±11.87 | 76.40±14.68 | 0.34 | 0.79 |
|  | T3 | 76.91±9.53 | 73.43±9.94 | 75.08±12.43 | 78.54±9.37 | 0.67 | 0.57 |
|  | T4 | 78.24±11.67 | 78.94±13.41 | 76.64±12.34 | 77.13±10.00 | 0.18 | 0.91 |
| **HR** | T0 | 81.52±16.10 | 72.67±11.58 | 75.05±10.22 | 66.00±4.00 | 2.11 | 0.11 |
|  | T1 | 71.88±10.47 | 69.47±10.41 | 72.95±10.83 | 65.57±7.72 | 1.20 | 0.31 |
|  | T2 | 69.86±10.62 | 66.32±9.36 | 70.39±8.99 | 63.25±8.41 | 1.87 | 0.14 |
|  | T3 | 67.51±11.69 | 64.45±9.73 | 67.00±9.04 | 64.29±5.22 | 0.56 | 0.64 |
|  | T4 | 67.72±9.83 | 66.71±7.37 | 65.14±8.08 | 67.14±16.96 | 0.51 | 0.68 |
| **SpO2** | T0 | 99.65±0.79 | 99.60±0.97 | 99.78±0.80 | 100.0±0.00 | 0.34 | 0.80 |
|  | T1 | 99.10±1.51 | 99.00±2.85 | 99.14±4.31 | 99.75±0.46 | 0.12 | 0.95 |
|  | T2 | 98.93±3.19 | 98.88±2.40 | 99.73±0.71 | 99.86±0.38 | 1.34 | 0.26 |
|  | T3 | 99.43±1.43 | 99.63±0.60 | 99.78±0.60 | 99.50±1.07 | 0.90 | 0.45 |
|  | T4 | 99.65±0.65 | 99.72±0.46 | 99.79±0.52 | 99.50±0.84 | 0.64 | 0.59 |
| **BIS** | T0 | 97.43±1.02 | 97.00±0082 | 97.29±9.62 | 97.00±0 | 0.42 | 0.74 |
|  | T1 | 58.23±11.36 | 55.90±7.047 | 56.76±13.46 | 57.17±13.26 | 0.13 | 0.94 |
|  | T2 | 60.40±5.06 | 57.17±7.18 | 57.58±8.81 | 57.13±7.97 | 1.07 | 0.37 |
|  | T3 | 60.85±5.96 | 59.55±5.61 | 58.78±9.09 | 59.11±6.09 | 0.42 | 0.74 |
|  | T4 | 61.54±5.885 | 61.20±4.686 | 60.31±8.627 | 60.80±8.121 | 0.16 | 0.92 |

SBP, systolic blood pressure; MBP, mean blood pressure; HR, heart rate; SpO2: Saturation of pulse oxygen; BIS, bispectral index.

**Supplementary 2. The mean dose of additional administration did not demonstrate statistical significance among the 4 groups.**

**Supplementary 3. Univariable linear regression analysis for induction dose as the dependent variable.**

| **Variables** | **Unstandardized Coefficients** | | **t** | **p** |
| --- | --- | --- | --- | --- |
|  | **B** | **Std.Error** |  |  |
| **Age** | -0.517 | 0.163 | -3.181 | **0.002** |
| **Gender** | -19.373 | 3.500 | -5.535 | **<0.001** |
| **Height** | 1.280 | 0.221 | 5.796 | **<0.001** |
| **Weight** | 0.853 | 0.134 | 6.386 | **<0.001** |
| **BMI** | 2.218 | 0.533 | 4.161 | **<0.001** |
| ASA classification | -3.942 | 5.517 | -0.714 | 0.476 |
| Chronic diseases | -6.110 | 6.429 | -0.951 | 0.343 |
| Other factors | 3.319 | 6.443 | 0.515 | 0.607 |
| **Anesthesia start time** | 2.197 | 0.776 | 2.832 | **0.005** |

**Supplementary 4. Collinearity analysis for the induction dose as the dependent variable.**

| **Variables** | **VIF** |
| --- | --- |
| Age | 1.155 |
| Gender | 2.518 |
| Height | 50.437 |
| Weight | 190.641 |
| BMI | 108.907 |
| ASA classification | 3.057 |
| Chronic diseases | 3.152 |
| Other disease that impact  propofol dose | 1.149 |
| Anesthesia start time | 1.052 |

**Supplementary 5. Univariable linear regression analysis for the dependent variable of maintenance dose per kilogram per hour.**

| **Variables** | **Unstandardized Coefficients** | | **t** | **p** |
| --- | --- | --- | --- | --- |
|  | **B** | **Std.Error** |  |  |
| Age | -0.021 | 0.017 | -1.216 | 0.226 |
| **Gender** | 0.882 | 0.394 | 2.237 | 0.027 |
| **Height** | -0.059 | 0.025 | -2.365 | 0.019 |
| **Weight** | -0.070 | 0.015 | -4.800 | <0.001 |
| **BMI** | -0.274 | 0.055 | -5.009 | <0.001 |
| ASA classification | -0.095 | 0.584 | -0.162 | 0.872 |
| Chronic diseases | -0.390 | 0.666 | -0.585 | 0.559 |
| **Other factors** | -1.143 | 0.659 | -1.734 | 0.085 |
| **Anesthesia start time** | 0.191 | 0.081 | 2.346 | 0.020 |
| **Induction dose per kilogram** | 1.144 | 0.564 | 2.029 | 0.044 |
| **Induction dose** | -0.014 | 0.009 | -1.644 | 0.102 |
| **Duration** | -0.083 | 0.041 | -2.037 | 0.044 |

**Supplementary 6. Collinearity analysis for the dependent variable of maintenance dose per kilogram per hour.**

| **Variables** | **VIF** |
| --- | --- |
| Age | 1.394 |
| Gender | 2.578 |
| Height | 60.557 |
| Weight | 194.880 |
| BMI | 127.186 |
| ASA classification | 3.539 |
| Chronic diseases | 3.551 |
| Other factors | 1.150 |
| Anesthesia start time | 1.133 |
| Induction dose per  kilogram | 19.898 |
| Induction dose | 22.801 |
| Duration | 1.256 |

**Supplementary 7. Models of outputs the induction dose and the maintenance per kilogram per hour**

| **Models** | **Variables** | | **RMSE** | **MAE** | **R^2^** |
| --- | --- | --- | --- | --- | --- |
| The induction  dose | | Age, BMI, gender, anesthesia start time, ASA classification, chronic disease, other factors | 21.81 | 15.17 | 0.13 |
|  |  | **Age, height, weight, gender, anesthesia start time, ASA classification, chronic disease, other factors** | **24.13** | **18.14** | **0.28** |
| The maintenance  dose per kilogram  per hour | | Age, height, weight, gender, anesthesia start time, ASA classification, duration, chronic diseases, other factors | 42.08 | 30.91 | 0.39 |
|  |  | **Age, BMI, gender, anesthesia start time, ASA classification, duration, chronic disease, other factors** | **1.61** | **1.15** | **0.37** |
|  |  | Age, BMI, gender, anesthesia start time, ASA classification, duration, chronic disease, other factors, induction dose | 1.64 | 1.11 | 0.34 |
|  |  | Age, BMI, gender, anesthesia start time, ASA classification, duration，chronic diseases, other factors, induction dose per weight | 1.74 | 1.19 | 0.26 |
|  |  | Age, height, weight, gender, anesthesia start time, ASA classification, duration, chronic diseases, other factors, induction dose per weight | 1.77 | 1.38 | 0.24 |

RMSE: Root mean square error; MAE: Mean absolute error; R^2^: the coefficient of determination

RMSE: Root mean square error; MAE: Mean absolute error; R^2^: the coefficient of determination


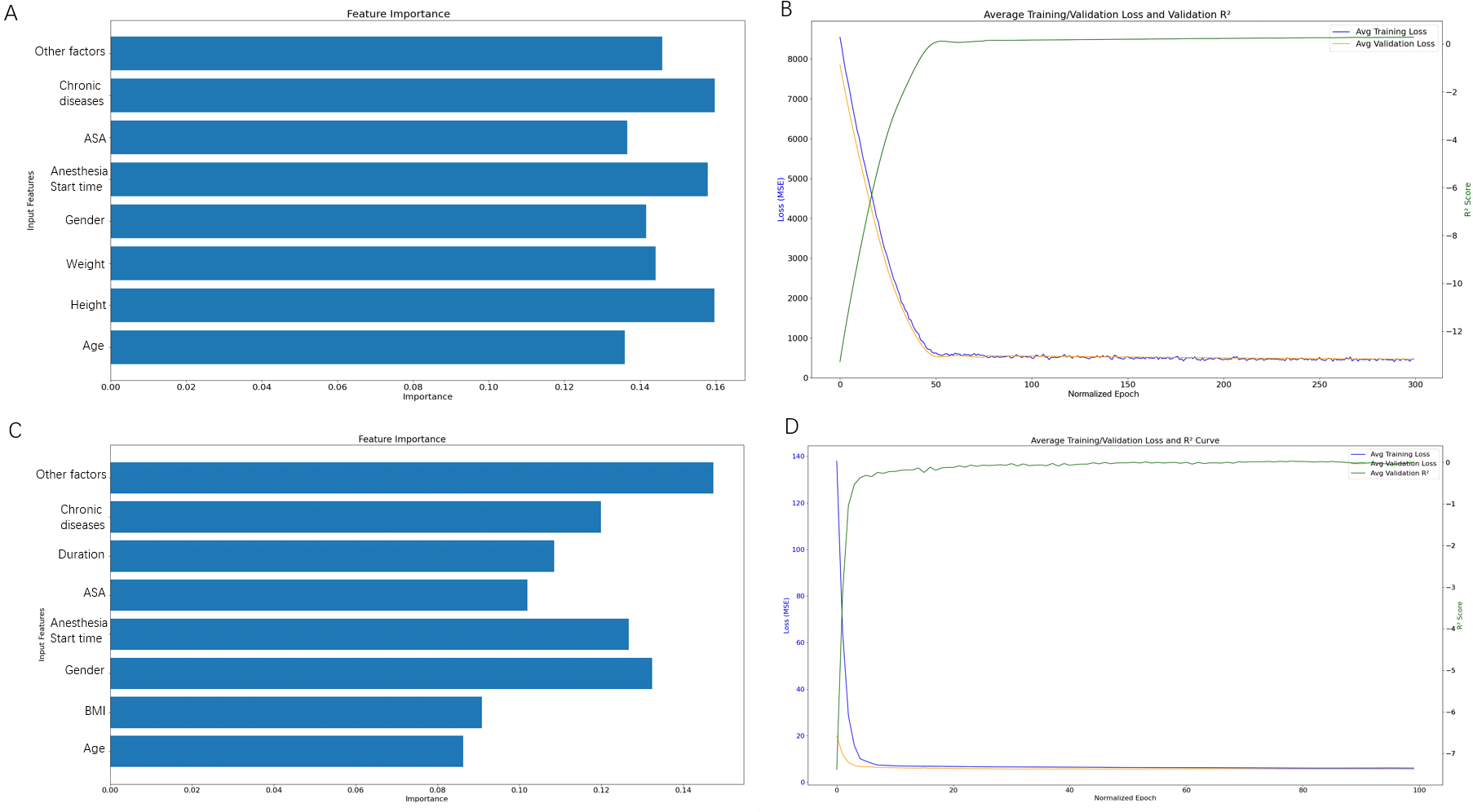


Supplementary 8. The feature importances and training/validation loss curves of the machine learning models. A. The feature importances of output Induction dose; B. The training loss, validation loss, and validation R² curves of output Induction dose; C. The feature importances of output Maintenance dose per kilogram per hour; D. The training loss, validation loss, and validation R² curves of output Maintenance dose per kilogram per hour.
